# The Role of Motor Learning on Measures of Physical Requirements and Motor Variability During Repetitive Screwing

**DOI:** 10.3390/ijerph16071231

**Published:** 2019-04-06

**Authors:** Tessy Luger, Robert Seibt, Monika A. Rieger, Benjamin Steinhilber

**Affiliations:** Institute of Occupational and Social Medicine and Health Services Research, University Hospital and University of Tübingen, Wilhelmstraße 27, 72074 Tübingen, Germany; robert.seibt@med.uni-tuebingen.de (R.S.); monika.rieger@med.uni-tuebingen.de (M.A.R.); benjamin.steinhilber@med.uni-tuebingen.de (B.S.)

**Keywords:** manual materials handling, electromyography (EMG), motor control, experience, electrocardiography, kinematics

## Abstract

We investigated whether physical requirements and motor variability decreased over days in novices during a repetitive screwing task. Fifty-seven subjects performed one hour of repetitive screwing and fastening on three days, separated by 2–7 days. The average physical requirement and relative cycle-to-cycle variability (coefficient of variation, i.e., CV) were calculated from continuous recordings of electromyography of four arm muscles (biceps brachii, triceps brachii, flexor carpi radialis, extensor digitorum), forearm acceleration, and electrocardiography. Muscle activity levels, heart rate, and forearm acceleration decreased from day 1 to day 2 (range: ~4% to ~20%) and/or 3 (range: ~4% to ~28%). Not all muscles showed a similar pattern. Activity of the extensor digitorum and biceps brachii decreased already between days 1 and 2 (range: ~6% to ~13%), whereas activity of the flexor carpi radialis and triceps brachii decreased between days 1 and 3 (range: ~13% to ~20%). No changes in physical requirement were detected between days 2 and 3. Relative motor variability did not change across days, except that variability of forearm acceleration increased from day 1 to 3 (~5%). This study found consistent changes in physical requirements and indicated that several arm muscles show earlier decreases of muscular activity, like the extensor digitorum, compared to other body parts, like the flexor carpi radialis. Moreover, movement strategies may develop differently than muscle activation strategies, based on the different developments of physical requirements and motor variability. The development of physical requirements in industrial tasks is part of daily living and starts at task onset, highlighting the importance of task familiarization and the randomization of experimental conditions in scientific studies.

## 1. Introduction

During our entire lives we acquire new skills, adapt to new situations, and learn to make decisions, which is known as “motor learning” [1]. Motor learning is an ongoing process and is initiated in movement preparations, during which our motor system explores the environment and the different possibilities to gradually improve motor control ("reinforcement learning") [2]. Performing a movement results from the synchronous interaction of multiple systems with the environment ("dynamic systems model") [3,4]. Within this dynamic systems model, our central nervous system (CNS) has to choose a pathway, from a vast number of movement options, to perform the action. In other words, our CNS has to overcome the “degrees of freedom (DOF) problem” and has to select one strategy to execute the desired movement [3]. This reflects the high redundancy of our motor system, with the plausible consequence that our actions become highly variable as well and that we are unable to repeat the exact same movement in repetitive occupational tasks. This phenomenon is termed motor variability, which has been defined as variance in movement of an individual who performs under similar task conditions [5].

The study of Madeleine et al. [6] illustrates that experienced workers showed more motor variability than novices. Sandlund et al. [7] even proposed that motor variability could be considered an individual or personal factor predicting which workers would be prone to develop work-related musculoskeletal disorders (WRMSD), theoretically implying that there could be an optimal level of motor variability. Several studies showed muscular activity levels and motor learning being negatively associated, meaning that muscular activity decreases along the process of motor learning in gaining experience [6,8]. Furthermore, muscular activity was higher when exposed to new environments compared to familiar environments [9,10].

In an occupational context, researchers and practitioners aim to design workplaces by means of ergonomics tools or work organization (e.g., task rotation) that lead to a decreased workload and reduce the risk of WRMSD. In this respect, the role of motor variability in relation to WRMSD has caught the attention of many researchers. Mainly, laboratory studies have been conducted to assess motor variability in an occupational context, since experienced workers from the field have limited availability and conducting field studies generally requires additional resources. Laboratory studies often include inexperienced subjects or novices to test what the effect of ergonomically improved tools or conditions is in a specific work simulation, judged by measures of physical requirements, work performance, and motor variability (e.g., [11,12]). Measures of physical requirements are the static, median or mean, and peak levels of muscular activity, according to the exposure variation analysis [13], median or mean of kinematic variables like joint angles, movement velocity or acceleration, and median or mean of the heart rate. Measures of motor variability are linear metrics like cycle-to-cycle standard deviation or coefficient of variation [14], or nonlinear metrics like entropy-based metrics and coordination metrics [15]. The difficulty of interpreting laboratory assessments among novices is that motor learning may influence study results and respective effects, expressed in terms of, e.g., muscular effort, could be misinterpreted. For example, early phases of the motor learning process could result in higher levels of muscle activity, as shown in a previous study [16].

The aim of this study is to investigate whether physical requirements and motor variability of novices, as a function of adjusted motor control strategies, change over a three-day repeated fine motor task. For investigating the physical requirements, we calculated the static (10th percentile), median (50th percentile), and peak (90th percentile) levels of muscular activity, the mean forearm acceleration, and the mean heart rate. For investigating motor variability, we calculated the cycle-to-cycle variability (coefficient of variation, CV) of muscular activity and forearm acceleration. We have used an exploratory approach because this study was originally not designed to investigate aspects of motor learning, but to assess the test-retest reliability of different normalization procedures of surface electromyography [17]. We hypothesized that measures of physical requirements and motor variability would decrease over days. With the results of this study, we aim to highlight methodological issues that should be considered when designing studies to minimize misinterpretation of results and increase practical relevance and whether we can rely on performing just one measurement to assess the risk of musculoskeletal disorders.

## 2. Materials and Methods 

### 2.1. Study Population

Subjects were excluded if they had any acute or cardiovascular diseases, impaired range of motion of the neck and upper extremities, or neurological impairments. A total of 65 subjects were recruited for study participation, of which 8 dropped-out due to methodological or organizational issues. The final study population consisted of 57 healthy subjects, of which 30 were female and 27 were male. Details with regard to the subjects can be found in Table 1. All subjects were inexperienced, meaning that none of them had specific experience in the repetitive task with the two specific screwing and fastening tools. The study (260/201BO2) was approved by the local ethics committee of the University of Tübingen (Germany) and all subjects signed the informed consent prior to participation.

### 2.2. Experimental Design

**Figure 1:**
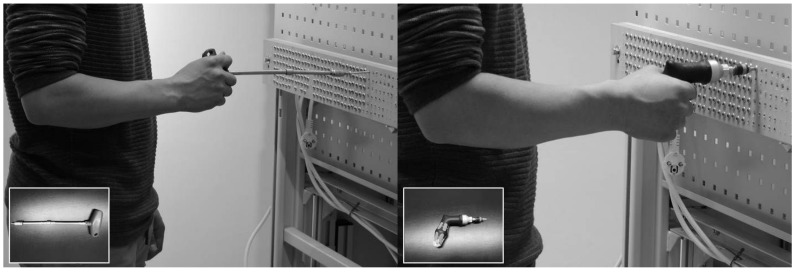
Experimental task setup with an example of screwing using the t-handle screwdriver (left) and an example of fastening using the torque screwdriver (right).

Subjects performed a repetitive screwing task with their dominant hand on a wooden plate that was positioned in front of them. The height of this plate was individually adjusted, so that the middle of the vertical screwing rows was aligned with the elbow height of the subjects while they were standing upright, with the hand palms facing anterior and the thumbs pointing lateral. For 60 min, subjects screwed 72 screws (4.5 × 45 mm, T-Start T20, 4 g, SPAX International GmbH & Co. KG, Ennepetal, Germany) into the wooden plate (multiplex, 30 mm thick), which was split into 12 vertical rows of six screws each. With their non-dominant hand the subjects positioned the 6 screws, taken from the container that was positioned close to their non-dominant hand, and screwed them in one after another with their dominant hand using a T-handle screwdriver (e.g., T-handle 336, T15, handle cross size 80 mm, shaft length 200 mm, 162 g incl. 3 g bit, Wiha, Germany; Figure 1), after which they pressed a buzzer (phase 1). Then, the subjects changed tools and fastened the same 6 screws with their dominant hand using a torque screwdriver (7443 pistol, 232 g incl. 3 g bit, Wera, Germany; Figure 1), after which they pressed a buzzer again (phase 2). These actions were repeated 12 times until all 12 vertical rows were filled with 6 screws. Due to using torx head screws, the amount of forward directed force was low. The torque screwdriver used for fastening released a click at a predetermined torque of 5 Nm. The buzzer was sampled along with the muscle activity recordings.

Subjects visited the laboratory on three days, separated by 2 to 7 days. At day 1, subjects performed for a period of ~10 min, during which they familiarized with, i.e., got acquainted with, the task. Subjects were considered familiar with the task when they were able to perform screwing and fastening with the two tools provided and work at the predetermined work pace. Following familiarization and at days 2 and 3, the subjects were prepared for the measurements, performed reference measurements for electromyographic (EMG) normalization, and performed the one-hour task.

The work pace of the subjects was controlled using visual feedback, i.e., a vertical bar representing a timeline showed the time that was left to fulfill screwing (phase 1) and fastening (phase 2) of each of the 12 rows. Work pace was predetermined based on the standardized, predetermined motion-time measurement system MTM [18], a system often used in industrial production planning. For both phases together we chose pace MTM-85 to allow all subjects to fulfill the task in the given time without developing muscular fatigue. This pace corresponded to 242 s for screwing (6 screws; phase 1) and 28 s for fastening (6 screws; phase 2), adding up to 270 s for one vertical row. Since we controlled the amount of work and the work pace and since we observed that all subjects screwed in the screws completely, we can conclude that performance was equal within subjects across days and also across subjects.

### 2.3. Experimental Design

#### 2.3.1. Muscular Activity

Electrical activities of the following four dominant superficial muscles of the hand-arm system that are involved in grasping and screwing, as verified in pilot measurements and as deduced from previous studies [19,20], were measured: The M. triceps brachii, M. biceps brachii, M. flexor carpi radialis, and M. extensor digitorum. Their electrical activity was recorded using surface EMG. We placed pre-gelled Ag/AgCl surface electrodes (35 × 26 mm, 15 mm active area diameter, Kendall^TM^ H93SG ECG Electrodes, Covidien, Zaltbommel, the Netherlands) in a bipolar configuration with an inter-electrode distance of 26 mm (center-to-center) on the muscle belly, according to Criswell [21]. A ground electrode, used to equalize the electrical ground level of the measurement device to the subject’s electrical level and to minimize electromagnetic interferences, was placed on the seventh cervical vertebra. Prior to electrode placement we shaved the skin, in case of body hair, and prepared it with an abrasive paste (Skin Prep Gel, Nuprep^®^, Aurora, USA).

EMG signals were differential amplified, analogue filtered (2nd order high pass filter, –3 dB at 4 Hz; 11th order low pass filter, –3 dB at 1300 Hz), sampled (4096 Hz), analyzed, and stored using a combined data analyzer and logger (PS11-UD, THUMEDI^®^ GmbH & Co. KG, Thum, Germany; overall CMRR > 96 dB; overall effective noise < 0.8 µV RMS; linearity type ±0.25 dB at 20–1100 Hz). The device real-time transformed the data into the frequency domain (1,024-point Fast Fourier Transformation, Bartlett-window, 50% overlap) and digitally high-pass filtered the signal (11^th^ order; –3 dB at 20 Hz). Power line interferences (50 Hz and its first seven harmonics) were removed by replacing it by the spectral values of a 4-Hz wide bandwidth around its center frequency by means of both spectral neighbors. The root-mean-square (RMS) of the electrical activity (μV) and median power frequency (MPF (Hz)) were real-time calculated from the power spectrum (250-millissecond moving window with 50 % overlap) and stored synchronously to the raw data by the PS11 device. An example recording of the RMS of the triceps brachii is provided in Figure S1 (Supplementary Material).

##### Normalization

Prior to the experimental task, we collected EMG during four 15-s submaximal reference voluntary contractions (RVC) with fixed force levels (Table 2). Each RVC targeted one of the four muscles, biceps brachii, triceps brachii, extensor digitorum, or flexor carpi radialis. The RVCs were measured using a self-developed measuring device in which the subject took a standardized position. With the upper body in an upright position, the forearm was placed horizontally and the upper arm perpendicular to the forearm. For determining the force of the biceps brachii and triceps brachii muscles, the force cell was positioned underneath the cushion below the distal end of the forearm. For determining the force of the extensor digitorum and flexor carpi radialis in the forearm, the force cell was positioned underneath the metacarpal bones in the hand. A monitor was connected to the force cell to give subjects visual feedback on the force level of the muscle contraction. Every RVC was followed by a recovery period of ~1 min. Electrical activity of the muscles was recorded during the RVCs and the middle 10 s of a steady-state period was used for EMG normalization. All RMS values were expressed as a percent of the electrical activity during the RVC, i.e., reference voluntary electrical activity (%RVE; Table 2), by dividing the experimental electrical activity level (μV) by the reference electrical activity level (μV) and multiplying by 100%.

##### Parameters

From the recordings of muscular activity, we determined the low or static level (10th percentile, RMS_10_), the median level (50th percentile, RMS_MEDIAN_), and the high level (90th percentile, RMS_90_) according to the amplitude probability distribution [22]. These parameters were determined for the overall task, screwing only (phase 1), and fastening only (phase 2). In addition, we calculated the within-day change of the MPF and RMS_MEDIAN_ to assess whether there were signs of electromyographic muscular fatigue, determined as an increased RMS_MEDIAN_ concomitant with a decreased MPF [23].

We calculated the relative cycle-to-cycle variability of RMS during screwing (phase 1) and fastening (phase 2) as a metric reflecting the size of motor variability. We defined one work cycle of screwing as screwing 1 screw (~40 s) and one work cycle of fastening as fastening 6 screws belonging to 1 vertical row (~28 s), resulting in 72 screwing and 12 fastening work cycles per day. The relative variability was assessed by calculating the RMS coefficient of variation (CV) per experimental day (RMS_CV_), i.e., the square root of the mean variance of RMS across screwing and fastening work cycles, divided by the RMS_MEAN_ of the screwing and fastening work cycles.

#### 2.3.2. Heart Rate

The electrical activity of the heart was recorded using electrocardiography (ECG) by two pre-gelled Ag/AgCl surface electrodes (35 × 26 mm, Kendall^TM^ H93SG ECG Electrodes, Covidien, Zaltbommel, the Netherlands) placed ~5 cm cranial and ~3 cm left-lateral from the distal end of the sternum and over the anterior to midaxillary line at the fifth left rib. The PS11 sampled (1000 Hz) and recorded the ECG signal (PS11-UD, THUMEDI^®^ GmbH & Co. KG, Thum, Germany), from which we calculated the mean heart rate (HR_MEAN_) for the overall task, for the screwing part (phase 1), and for the fastening part (phase 2).

#### 2.3.3. Forearm Acceleration

Acceleration of the forearm was sampled (4096 Hz) and recorded (PS11-UD, THUMEDI^®^ GmbH & Co. KG, Thum, Germany) using a single-axis accelerometer (resolution 0.005 m/s^2^) placed ventral over the extensor digitorum, 2 to 3 cm ventral of the styloid process ulnaris, using double-sided adhesive tape. Its orientation, i.e., its sensitive axis, was the orthogonal of the forearm bones ulna and radius. The position and orientation were chosen to avoid mechanical interference with the EMG recording electrodes. The accelerometer measured the flexion-extension and rotational acceleration of the forearm. The RMS of the acceleration data was real-time calculated (250-millissecond moving window with 50% overlap), from which we determined the mean (ACC_MEAN_) for the overall task, screwing only (phase 1), and fastening only (phase 2). Similar as for muscular activity, we calculated the relative cycle-to-cycle variability of the forearm acceleration (ACC_CV_), for each experimental day, by dividing the square root of the mean variance across all work cycles for screwing and fastening by the ACC_MEAN_.

### 2.4. Statistical Analysis

We checked whether the parameters were normally distributed by visually inspecting the histograms and skewness and kurtosis values [24,25]. Based on these explorations, RMS_10_, RMS_MEDIAN_, RMS_90_, and RMS_CV_ were not normally distributed and were therefore log-transformed before the statistical analyses. We performed repeated-measures analysis of variance (RM-ANOVA) on the mean parameters (i.e., RMS_10_, RMS_MEDIAN_, RMS_90_, HR_MEAN_, and ACC_MEAN_) of the overall one-hour task, with day as the within-subject factor. We also performed RM-ANOVA (day as within-subject factor) on the mean parameters of phases 1 and 2 separately (i.e., RMS_10_, RMS_MEDIAN_, RMS_90_, RMS_CV_, HR_MEAN_, ACC_MEAN_, and ACC_CV_). In case of a significant main effect of day, we performed post hoc tests using Student’s T-Tests with Bonferroni correction and calculated Cohen’s *d* effect sizes using the pooled standard deviation of the two respective days as a standardizer [26]. All statistical analyses were performed with JMP (JMP^®^ 13.1.0) and statistical significance was accepted for the main effects when *p* < 0.05 or, for the Bonferroni-corrected post hoc comparisons, when *p* < 0.0167 (i.e., 0.05 divided by three comparisons). Effect sizes were considered small (*d* ≥ 0.2), medium (*d* ≥ 0.5), or large (*d* ≥ 0.8), as suggested by Cohen [27].

## 3. Results

All data with statistical outcomes are summarized in Table S1 (Supplementary Material). Data of a different number of subjects was available for each parameter due to failed or unreliable recordings, of which the specific number of subjects is mentioned in Table S1 (Supplementary Material).

### 3.1. Muscular Fatigue

None of the muscles showed signs of muscular fatigue. Only the flexor carpi radialis showed a significant decrease in RMS_MEDIAN_ concomitant with a significant increase in MPF, which actually points towards the opposite of muscular fatigue, i.e., recovery of muscular strength [23].

### 3.2. Muscular Activity

#### 3.2.1. M. Triceps Brachii

**Figure 2:**
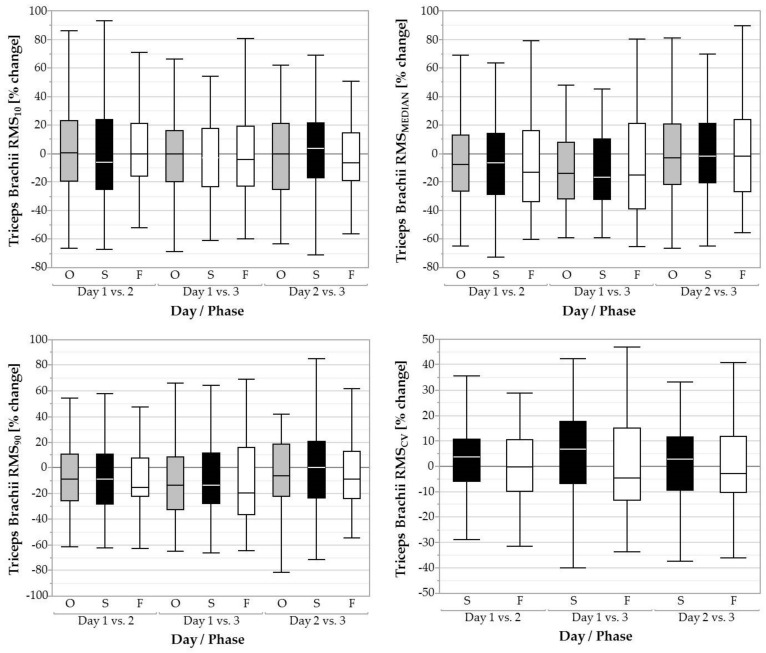
Boxplots of the 10th, 50th, and 90th percentile muscular activity (RMS_10_, RMS_MEDIAN_, RMS_90_) and coefficient of variation (RMS_CV_) of the triceps brachii over three days for the overall task (O, grey filled boxplots), the screwing phases (S, black filled boxplots), and the fastening phases (F, white filled boxplots).

The factor day had a significant effect on RMS_MEDIAN_ and RMS_90_ during the overall task (*F* = 8.88, *p* < 0.001), the screwing phase (*F* = 7.64, *p* < 0.001), and the fastening phase (*F* = 5.81, *p* < 0.01). For the overall task, RMS_MEDIAN_ decreased from day 1 (11.80 ± 9.56 %RVE) to day 2 (10.31 ± 8.33 %RVE; *p* < 0.01, *d* = 0.16) and from day 1 (11.80 ± 9.56 %RVE) to day 3 (9.32 ± 6.84 %RVE; *p* < 0.001, *d* = 0.30). For screwing, RMS_MEDIAN_ decreased from day 1 (13.74 ± 11.12 %RVE) to day 2 (12.08 ± 10.17 %RVE; *p* < 0.01, *d* = 0.16) and from day 1 (13.74 ± 11.12 %RVE) to day 3 (10.98 ± 9.18 %RVE; *p* < 0.001, *d* = 0.29). For fastening, RMS_MEDIAN_ decreased from day 1 (10.23 ± 7.97 %RVE) to day 3 (8.23 ± 6.05 %RVE; *p* < 0.01, *d* = 0.29). RMS_90_ decreased in the overall task (*F* = 5.27, *p* < 0.01) from day 1 (38.03 ± 26.13 %RVE) to day 3 (30.82 ± 17.73 %RVE; *p* < 0.01, *d* = 0.33), in the screwing phase also (*F* = 4.38, *p* < 0.05) from day 1 (38.25 ± 26.20 %RVE) to day 3 (31.74 ± 18.83 %RVE; *p* < 0.01, *d* = 0.29), and in the fastening phase (*F* = 9.09, *p* < 0.001) from day 1 (48.47 ± 36.00 %RVE) to day 2 (31.18 ± 31.80 %RVE; *p* < 0.01, *d* = 0.16) and from day 1 (48.47 ± 36.00 %%RVE) to day 3 (39.26 ± 25.81 %RVE; *p* < 0.001, *d* = 0.30; Figure 2).No changes in RMS_10_ or RMS_CV_ were found between days.

#### 3.2.2. M. Biceps Brachii

**Figure 3:**
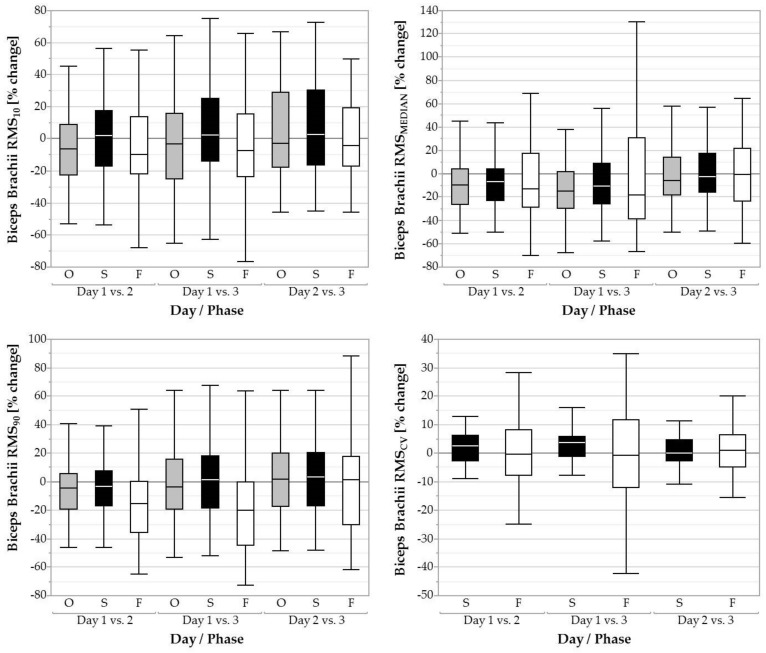
Boxplots of the 10th, 50th, and 90th percentile muscular activity (RMS_10_, RMS_MEDIAN, RMS_90) and coefficient of variation (RMS_CV_) of the biceps brachii over three days for the overall task (O, grey filled boxplots), the screwing phases (S, black filled boxplots), and the fastening phases (F, white filled boxplots).

RMS_MEDIAN_ of the overall task was significantly influenced by day (*F* = 5,89, *p* < 0.01), with day 1 holding higher values (28.83 ± 15.01 %RVE) than day 2 (25.51 ± 13.82 %RVE; *p* < 0.01, *d* = 0.23) and day 3 (25.50 ± 13.60 %RVE; *p* < 0.01, *d* = 0.23; Figure 3). RMS_MEDIAN_ for the screwing phase also showed a significant effect of day (*F* = 3.25, *p* < 0.05) but post hoc tests revealed no significant pairwise comparisons. RMS_90_ was significantly influenced by day during fastening (F = 14.64, p < 0.001) with a decrease from day 1 (148.33 ± 69.01 %RVE) to 2 (122.52 ± 62.60 %RVE; *p* < 0.001, *d* = 0.36) and also from day 1 (148.33 ± 69.01 %RVE) to 3 (114.85 ± 58.87 %RVE; *p* < 0.001, *d* = 0.49). RMS_10_ and RMS_CV_ did not change between days.

#### 3.2.3. M. Flexor Carpi Radialis

**Figure 4:**
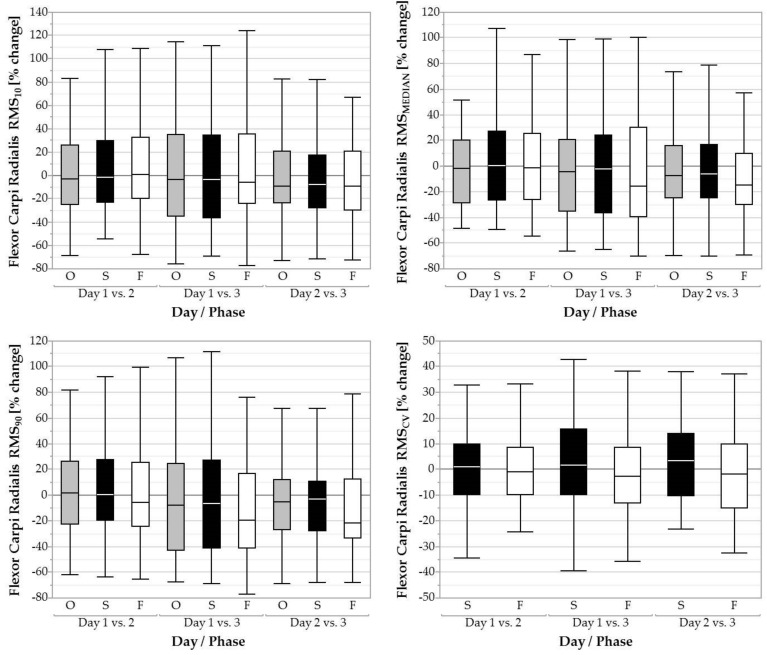
Boxplots of the 10th, 50th, and 90th percentile muscular activity (RMS_10_, RMS_MEDIAN, RMS_90) and coefficient of variation (RMS_CV_) of the flexor carpi radialis over three days for the overall task (O, grey filled boxplots), the screwing phases (S, black filled boxplots), and the fastening phases (F, white filled boxplots).

During the fastening phase, RMS_90_ was significantly influenced by day (*F* = 4.09, *p* < 0.05; Figure 4). Post hoc tests showed a decreased RMS_90_ from day 1 (65.90 ± 80.80 %RVE) to 3 (52.52 ± 53.75 %RVE; *p* < 0.01, *d* = 0.20). No significant difference between days was found for RMS_10_, RMS_MEDIAN,_ or RMS_CV_.

#### 3.2.4. M. Extensor Digitorum

**Figure 5:**
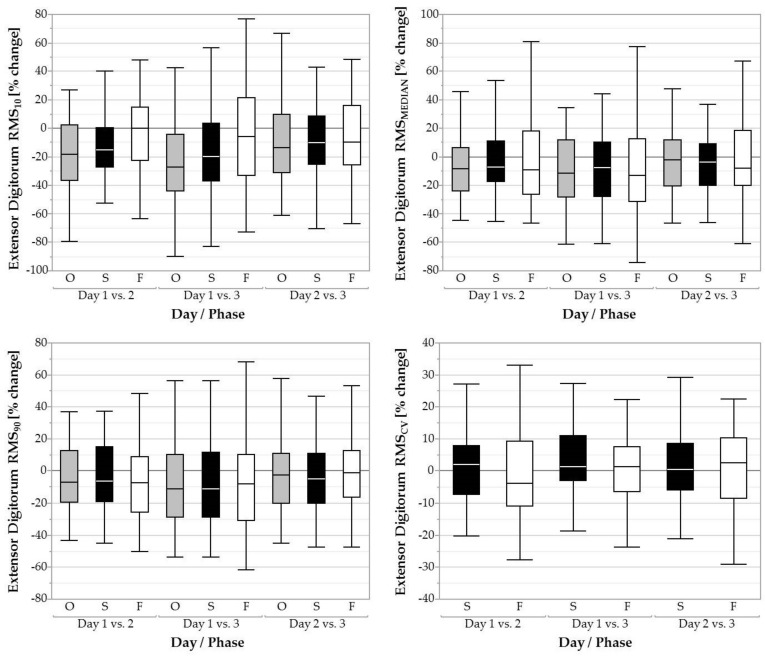
Boxplots of the 10^th^, 50^th^,and 90^th^ percentile muscular activity (RMS_10_, RMS_MEDIAN_, RMS_90_) and coefficient of variation (RMSV) of the extensor digitorum over three days for the overall task (O, grey filled boxplots), the screwing phases (S, black filled boxplots), and the fastening phases (F, white filled boxplots).

We found a significant main effect of day for RMS_MEDIAN_ in the overall task (*F* = 8.56, *p* < 0.001), during screwing (*F* = 6.32, *p* < 0.01), and during fastening (*F* = 3.31, *p* < 0.05). During the overall task, RMS_MEDIAN_ decreased from day 1 (45.29 ± 22.23 %RVE) to 2 (42.51 ± 23.57 %RVE; *p* < 0.05, *d* = 0.12) and from day 1 (45.29 ± 22.23 %RVE) to 3 (40.46 ± 22.00 %RVE; *p* < 0.001, *d* = 0.22). RMS_MEDIAN_ decreased from day 1 (50.34 ± 25.56 %RVE) to 3 (46.00 ± 26.25 %RVE) during screwing (*p* < 0.001, *d* = 0.17) and decreased from day 1 (34.76 ± 18.44 %RVE) to 3 (31.76 ± 19.19 %RVE) during fastening (*p* < 0.05, *d* = 0.16). RMS_10_ differed significantly between days for the overall task (*F* = 18.35, *p* < 0.001), screwing (*F* = 13.72, *p* < 0.001), and fastening (*F* = 3.50, *p* < 0.05; Figure 5). In the overall task, RMS_10_ decreased from day 1 (12.70 ± 7.06 %RVE) to 2 (10.13 ± 6.59 %RVE; *p* < 0.001, *d* = 0.38) and from day 1 (12.70 ± 7.06 %RVE) to 3 (9.12 ± 5.84 %RVE; *p* < 0.001, *d* = 0.55). In the screwing phase, RMS_10_ decreased from day 1 (17.45 ± 9.45 %RVE) to 2 (15.03 ± 8.73 %RVE; *p* < 0.01, *d* = 0.27) and from day 1 (17.45 ± 9.45 %RVE) to 3 (14.04 ± 8.07 %RVE; *p* < 0.001, *d* = 0.39). In the fastening phase, RMS_10_ decreased from day 1 (16.43 ± 9.30 %RVE) to 3 (15.18 ± 10.42 %RVE; *p* < 0.05, *d* = 0.13). The factor day also significantly influenced RMS_90_ for the overall task (*F* = 5.96, *p* < 0.01), screwing (*F* = 5.24, *p* < 0.01), and fastening (*F* = 4.27, *p* < 0.05). RMS_90_ decreased from day 1 (83.00 ± 45.24 %RVE) to 3 (75.60 ± 43.64 %RVE) in the overall task (*p* < 0.001, *d* = 0.17), decreased from day 1 (84.22 ± 47.10 %RVE) to 3 (77.29 ± 45.61 %RVE) in the screwing phase (*p* < 0.01, *d* = 0.15) and decreased from day 1 (84.14 ± 38.57 %RVE) to 3 (76.82 ± 38.52 %RVE) in the fastening phase (*p* < 0.01, *d* = 0.19). No significant effect of day on RMS_CV_ was found.

### 3.3. Heart Rate

**Figure 6:**
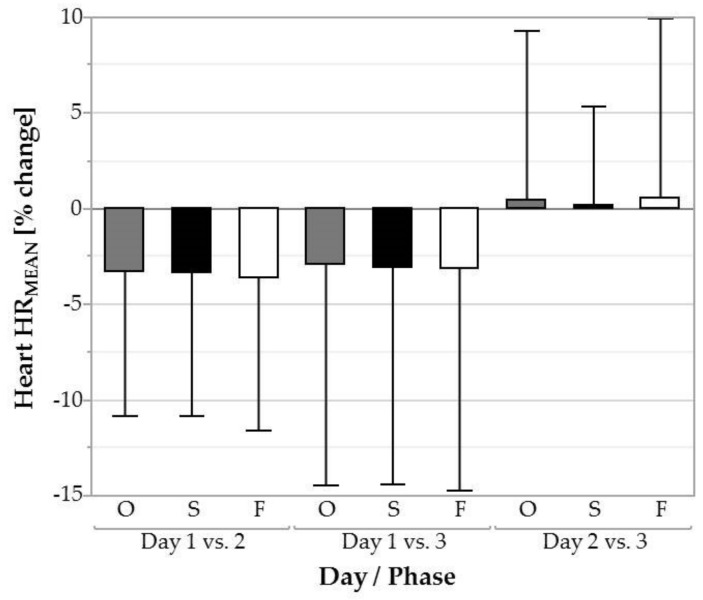
Average heart rate (HR_MEAN_) over the three days for the overall task (O, grey filled plots), the screwing phases (S, black filled plots), and the fastening phases (F, white filled plots). Error bars represent the SD across subjects.

HR_MEAN_ differed significantly between days (Figure 6) for the overall task (*F* = 5.91, *p* < 0.01), screwing (*F* = 9.38, *p* < 0.001), and fastening (*F* = 6.35, *p* < 0.01). For the overall task, HR_MEAN_ decreased from day 1 (91 ± 15 bpm) to 2 (88 ± 12 bpm; *p* < 0.01, *d* = 0.27) and from day 1 (91 ± 15 bpm) to 3 (88 ± 11 bpm; *p* < 0.01, *d* = 0.28). For screwing, HR_MEAN_ decreased also from day 1 (92 ± 15 bpm) to 2 (88 ± 12 bpm; *p* < 0.001, *d* = 0.27) and from day 1 (92 ± 15 bpm) to 3 (88 ± 11 bpm; *p* < 0.001, *d* = 0.29). For fastening it decreased from day 1 (89 ± 15 bpm) to 2 (85 ± 12 bpm; *p* < 0.01, *d* = 0.28) and from day 1 (89 ± 15 bpm) to 3 (85 ± 12 bpm; *p* < 0.01, *d* = 0.29).

### 3.4. Forearm Acceleration

**Figure 7:**
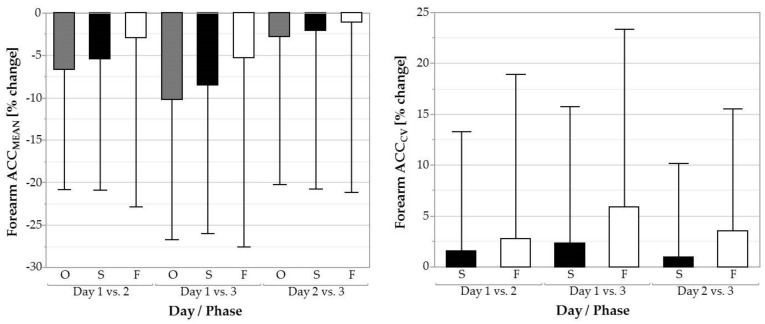
Average acceleration (ACC_MEAN_) and coefficient of variation (ACC_CV_) of the forearm over three days for the overall task (O, grey filled plots), the screwing phases (S, black filled plots), and the fastening phases (F, white filled plots). Error bars represent the SD across subjects.

ACC_MEAN_ significantly decreased over days in the overall task (*F* = 13.66, *p* < 0.001) and over the screwing phases (*F* = 9.10, *p* < 0.001; Figure 7). For the overall task, ACC_MEAN_ decreased from day 1 (237.13 ± 56.34 mm/s^2^) to 2 (218.65 ± 51.36 mm/s^2^; *p* < 0.001, *d* = 0.34) and from day 1 (237.13 ± 56.34 mm/s^2^) to 3 (209.45 ± 49.66 mm/s^2^; *p* < 0.001, *d* = 0.52). For screwing, ACC_MEAN_ decreased also from day 1 (261.84 ± 65.51 mm/s^2^) to 2 (244.35 ± 60.95 mm/s^2^; *p* < 0.01, *d* = 0.28) and from day 1 (261.84 ± 65.51 mm/s^2^) to 3 (235.34 ± 58.49 mm/s^2^; p < 0.001, d = 0.43). We found a main effect of day for ACC_CV_ during fastening (*F* = 3.59, *p* < 0.05). Post hoc tests revealed that ACC_CV_ significantly increased from day 1 (1.52 ± 0.29) to day 3 (1.61 ± 0.36; *p* < 0.01, *d* = −0.25).

## 4. Discussion

The objective of this study was to investigate whether physical requirements and motor variability decreased over three days of repetitive screwing among novices. The results largely support our hypothesis, showing a decrease over days in the static, median, and peak EMG activity levels for the extensor digitorum and biceps brachii, but to a lesser extent for the flexor carpi radialis and triceps brachii. No significant differences in physical requirements between days 2 and 3 were detected. Similarly, acceleration of the forearm and heart rate decreased over days. We found no support for the hypothesis that relative cycle-to-cycle variability of the muscles decreased over days, whereas the relative variability of forearm acceleration significantly increased over days.

### 4.1. Biomechanical and Cardiovascular Control Strategies

The simulated screwing task activated several upper and lower arm muscles, of which we have measured only a selection. It hereby appeared that the biceps brachii produced the highest activity levels as can be indicated from the normalized EMG values, which relates to its main function of lower arm supination [28] and the T -handle torx screwdriver not requiring much grip force.

The extensor digitorum and triceps brachii clearly decreased their median and peak muscle activity. Similar findings were reported by previous studies that found reductions in muscular activity as a result of motor learning during training (e.g., [29,30,31]). The overall lowered muscular activity production for the same occupational task may indicate that the early stage of motor learning is a process at the level of the central nervous system, which is supported by the decreased heart rate of ~4 bmp (similar to ~4%) over days, since a decreased heart rate reflects less inhibited parasympathetic activation of the autonomic nervous system [32].

Observing the behaviour of all four muscles separately made us conclude that not all behaved similarly over the three days. The flexor carpi radialis and biceps brachii mainly contributed to the fastening phase of the experimental task, since their peak muscular activity levels decreased between day 1 and days 2 and 3, especially during fastening. On the other hand, the triceps brachii and extensor digitorum showed the most prominent changes during the overall task, as indicated by their decreased median and peak activity levels from day 1 to days 2 and 3. These results indicate that the flexor muscles became more efficient, especially during fastening, and may benefit more from specific training than the extensor muscles that showed less specific changes between the three days.

The strong decreased forearm acceleration after the first day, especially during screwing, could point to a more efficient motor program that has significantly developed already after one day. The resulting movement patterns of screwing being smoother on days 2 and 3 may have resulted in more muscle relaxation, as reflected by the decreased static muscular activity level of the extensor digitorum. Note that all changes in physical requirements were detected at day 2 or 3, compared to day 1. This may indicate that the first day is highly important for developing motor control strategies, whereas no significant changes or improvements happen between days 2 and 3.

### 4.2. Motor Variability

The amount of motor variability of muscular activity levels remained equal over days in this study, which could imply that the screwing task was not new enough or too simple to provoke developments in motor variability, or that the follow-up was not long enough to be able to find significant developments. The relative variability of forearm acceleration, on the other hand, showed a slight but significant increase regarding the first two days (rate of ~7% increase). This strongly implies that movement strategies and muscular activation strategies develop in a different way. As suggested by previous studies, motor control strategies are optimized with increasing work experience, which could evoke an increased motor variability as a strategy to adapt to performance constraints such as muscular fatigue [33,34] and acute or chronic pain [35].

### 4.3. Practical Implications

#### 4.3.1. Importance of Familiarization and Randomization

This study with a within-subject design showed decreased muscular activity and acceleration patterns among novices who gain experience in performing a simple screwing task repeated over three days. Decreases were most prevalent between day one and the other two days but were absent between days 2 and 3. This finding indirectly highlights the importance of both sufficient familiarization to experimental tasks and randomization of experimental conditions in studies. Familiarization is especially important when designing a study including repeated measures, to decrease the influence of early stages in the motor learning process. However, it is not clear how much familiarization time is needed, as this probably highly depends on the task complexity. For example, in a study among novice and expert butchers, Madeleine, et al. [36] showed that motor strategies are in development during a six-month follow-up. This does not mean that follow-up times or familiarization periods should be equal to or longer than six months, because this is unrealistic to strive for in studies. However, familiarization should not be too short and include at least some physical practice. Based on the current study results, a two-minute familiarization period, as used by Qin et al. [37], might be too short to reliably measure and interpret physical requirements of occupational tasks. Not reporting task familiarization at all, like Wang et al. [38] did, makes the reader assume that familiarization was not part of the experimental protocol at all and could mean that outcomes were influenced by early stages of the motor learning process. Since the first day of this repetitive task seemed to be most prominent in developing motor control strategies, researchers should provide enough familiarization when investigating occupational tasks.

For comparing different experimental conditions, one can use a between-subject design or a within-subject design. In a between-subject design, classically, subjects are randomly assigned to a group (control vs. intervention). In a within-subject design, subjects will perform all experimental conditions (cross-over design) and are randomly assigned to a sequence of experimental conditions. In most cases, the randomization is performed counterbalanced, meaning that first the number of sequences or the number of subjects per group is determined, after which the assignment to one of the conditions or sequences is based on randomization. The well-known reasons to apply randomization in studies are twofold as follows: (1) Decreasing systematic errors created by a specific experimental manipulation [39] and (2) increasing generalizability or external validity [40]. In addition, we emphasize that randomization may also decrease effects of lack of or insufficient familiarization. This means that an over- or underestimation of the original research question is equally distributed across conditions or across intervention groups. When having a close look at the recent conference proceedings of the International Ergonomics Association 2018 [41], only six of the 223 short papers published, mention randomization in their study. This emphasizes that few studies take randomization into consideration and, as a result, the quality of the study and, therefore, also the relevance of the outcomes may decrease. We therefore recommend applying randomization when possible and, especially when recruiting novices (e.g., students), including a familiarization phase to decrease most of potential motor learning effects.

#### 4.3.2. Motor Variability in An Occupational Context

This study assessed the functional development of motor variability in a manual materials handling task over three days. Although we could not find changes in motor variability over three days, previous studies showed that experienced workers show more variable movement patterns [6], which may indicate that experienced workers are already in the final improvement stage of motor learning where they have developed movement behaviour enabling them to adapt to environmental constraints [42,43]. Furthermore, motor variability has also been suggested to play a beneficial role with respect to internal constraints, such as muscular fatigue [33], pain [44], and external constraints, such as task precision and pace [45]. Confounding factors are also suggested to play a role in the association between motor variability and internal/external constraints, such as sex [46], age [47], and health status. Our current knowledge about the role of motor variability in relation to the aforementioned concepts is still limited. However, when we continue studying motor variability in occupational settings, we might be able to (1) identify workers who are more prone to develop musculoskeletal disorders than others and (2) design (individually adjusted) work tasks and workstations in such a way that motor variability can be promoted [48].

### 4.4. Methodological Considerations

Our study supports the hypothesis that muscular activity decreased over three days of repetitive screwing and fastening among novices, although it should be noted that our sample size was rather large due to the exploratory design, which is usually not suitable to examine a hypothesis. In general, effect sizes of the reported results were rather small and only few results were accompanied by moderate effect sizes (*d* = 0.50), including the high muscular activity level of the biceps brachii, the static activity level of the extensor digitorum, and forearm acceleration. Muscular co-contraction has been related to aspects of motor learning in a previous study, [16], by calculating a complex index including the muscular activity and torque levels around a joint, which was beyond the scope of this study.

## 5. Conclusions

This study showed that physical requirements start developing at task onset, as reflected by the measures of muscular activity, acceleration, and heart rate. However, not all of these measures showed a similar pattern over days, i.e., the extensor digitorum and biceps brachii already showed changes between days 1 and 2, whereas the flexor carpi radialis and triceps brachii showed changes between days 1 and 3. This may indicate the more prominent role of both arm extensor muscles in the screwing and fastening task, regarding the earlier changes. However, changes between days 2 and 3 in physical requirements were absent. Motor variability of the selected muscles did not change between days, but variability of forearm acceleration increased from day 1 to 3, which may reflect that movement strategies develop differently than muscular activation strategies. We emphasize that these developments are part of daily life and should be considered when designing and interpreting studies in terms of task familiarization and randomization of experimental conditions.

## Figures and Tables

**Table 1:** Description of the study population, as a whole and broken down by sex. Results are displayed as value or as mean ± standard deviation (SD).

	Whole Population	Females	Males
Study population [N]	57	30	27
Age [years]	34.8 ± 14.0	36.5 ± 14.9	32.9 ± 13.0
Height [cm]	174.3 ± 8.9	167.4 ± 5.2	181.9 ± 5.1
Weight [kg]	73.3 ± 13.5	66.2 ± 9.8	81.1 ± 12.8
Handedness [N left/N right]	4/53	1/29	3/24
Sport [hours/week]	5.2 ± 4.5	3.9 ± 2.3	6.8 ± 5.8

**Table 2:** Descriptions of submaximal reference voluntary contractions (RVC) of the four target muscles, with corresponding force levels at which subjects had to sustain the RVC.

Target muscle	RVC Procedure	Force Level [N] ^1^
M. triceps brachii	Producing a downward force, elbow extension.	80
M. biceps brachii	Producing an upward force, elbow flexion.	110
M. flexor carpi radialis	Producing an upward force, wrist flexion.	60
M. extensor digitorum	Producing a downward force, wrist extension.	60

^1^ The force levels [N] were determined during pilot measurements among five subjects to correspond moderate force levels of ~40% maximal force.

## Supplementary Materials

The following are available online at https://www.mdpi.com/1660-4601/16/7/1231/s1, Figure S1: Example of a recording of the electrical activity of the triceps brachii of subject 823. The graphs show the raw RMS signal for day 1 (upper), day 2 (middle), and day 3 (lower); Table S1: Results of the one-way repeated measures analysis of variance with the within-subject factor ‘day’.
