# Supplementary material for: The Role of Motor Learning on Measures of Physical Requirements and Motor Variability During Repetitive Screwing
Source: Int J Environ Res Public Health. 2019 Apr 6;16(7):1231. doi: 10.3390/ijerph16071231 (PMC6479693; doi:10.3390/ijerph16071231)
Supplement: Supplementary file 1 [file ijerph-16-01231-s001.zip › Table_S_for_Supplementary_Material_REVISION.docx]

Table S1. Results of the one-way repeated measures analysis of variance with the within-subject factor ‘day’. Post hoc tests are performed using Student’s T-Tests with Bonferroni correction and Cohen’s d effect sizes are calculated. Statistical significance for the main factor is accepted when p < 0.05 and for the post hoc pairwise comparisons when p < 0.0167. The non-transformed mean and standard deviations (Mean ± SD) are provided; the non-transformed mean differences (MD) between days are provided with the post hoc pairwise comparisons. Muscle activities are expressed as percent of the electrical activity during the RVC, i.e. reference voluntary electrical activity (%RVE); heart rate is expressed in beats per minute (bpm); acceleration is expressed as mm per second squared (mm/s^2^).

| **Muscle / Body part** | **Parameter** | **Task** | **Subjects**  **N** | **Day 1** | **Day 2** | **Day 3** | **Main factor: day** | | **Post hoc tests** | | | | | | | | |
| --- | --- | --- | --- | --- | --- | --- | --- | --- | --- | --- | --- | --- | --- | --- | --- | --- | --- |
|  |  |  |  |  |  |  |  |  | **Day 1 vs. 2** | | | **Day 1 vs. 3** | | | **Day 2 vs. 3** | | |
|  |  |  |  | **Mean ± SD** | **Mean ± SD** | **Mean ± SD** | ***F*-value** | ***p*-value** | **MD** | ***p*-value** | **Cohen’s *d*** | **MD** | ***p*-value** | **Cohen’s *d*** | **MD** | ***p*-value** | **Cohen’s *d*** |
| M.  triceps  brachii | RMS_MEDIAN_  [%RVE] | Overall | 55 | 11.80 ± 9.56 | 10.31 ± 8.33 | 9.32 ± 6.84 | **8.8839** | **0.0003** | **1.49** | **0.0048** | **0.2018** | **2.47** | **< 0.0001** | **0.3031** | 0.98 | 0.2239 | 0.0903 |
|  |  | Screwing | 55 | 13.74 ± 11.12 | 12.08 ± 10.17 | 10.98 ± 9.18 | **7.6377** | **0.0008** | **1.66** | **0.0053** | **0.2105** | **2.76** | **0.0003** | **0.2937** | 0.10 | 0.3711 | 0.0699 |
|  |  | Fastening | 55 | 10.23 ± 7.97 | 9.06 ± 6.81 | 8.23 ± 6.05 | **5.8109** | **0.0040** | 1.17 | 0.0271 | 0.1776 | **1.33** | **0.0011** | **0.2804** | 0.83 | 0.2720 | 0.0927 |
|  | RMS_10_  [%RVE] | Overall | 55 | 3.29 ± 2.25 | 3.30 ± 2.35 | 3.07 ± 2.16 | 0.7417 | 0.4787 | - | - | - | - | - | - | - | - | - |
|  |  | Screwing | 55 | 3.91 ± 2.70 | 3.84 ± 2.73 | 3.62 ± 2.43 | 1.0426 | 0.3561 | - | - | - | - | - | - | - | - | - |
|  |  | Fastening | 55 | 4.67 ± 3.62 | 4.70 ± 3.50 | 4.34 ± 3.31 | 1.0804 | 0.3431 | - | - | - | - | - | - | - | - | - |
|  | RMS_90_  [%RVE] | Overall | 55 | 38.03 ± 26.13 | 36.81 ± 31.86 | 30.82 ± 17.73 | **5.2690** | **0.0066** | 1.21 | 0.1648 | 0.1184 | **7.21** | **0.0016** | **0.2968** | 6.00 | 0.0688 | 0.1650 |
|  |  | Screwing | 55 | 38.25 ± 26.20 | 36.90 ± 36.39 | 31.74 ± 18.83 | **4.3768** | **0.0149** | 1.34 | 0.0943 | 0.1377 | **6.50** | **0.0039** | **0.2563** | 5.16 | 0.2102 | 0.1086 |
|  |  | Fastening | 55 | 48.47 ± 36.00 | 31.18 ± 31.80 | 39.26 ± 25.81 | **9.0912** | **0.0002** | **5.29** | **0.0143** | **0.1817** | **9.21** | **< 0.0001** | **0.3114** | 3.92 | 0.0826 | 0.1283 |
|  | RMS_CV_ | Screwing | 55 | 0.91 ± 0.31 | 0.94 ± 0.32 | 0.94 ± 0.31 | 0.9193 | 0.4019 | - | - | - | - | - | - | - | - | - |
|  |  | Fastening | 55 | 1.07 ± 0.26 | 1.07 ± 0.26 | 1.07 ± 0.27 | 0.0565 | 0.9451 | - | - | - | - | - | - | - | - | - |
| M.  biceps  brachii | RMS_MEDIAN_  [%RVE] | Overall | 55 | 28.83 ± 15.01 | 25.51 ± 13.82 | 25.50 ± 13.60 | **5.8865** | **0.0037** | **3.88** | **0.0068** | **0.2824** | **3.19** | **0.0021** | **0.3039** | 0.70 | 0.7025 | 0.0369 |
|  |  | Screwing | 55 | 41.41 ± 19.31 | 37.82 ± 17.46 | 38.41 ± 18.57 | **3.2486** | **0.0427** | 3.28 | 0.0316 | 0.2179 | 1.75 | 0.0274 | 0.2115 | 1.53 | 0.9527 | 0.0056 |
|  |  | Fastening | 55 | 20.08 ± 14.28 | 17.42 ± 14.04 | 18.31 ± 14.45 | 2.8676 | 0.0612 | - | - | - | - | - | - | - | - | - |
|  | RMS_10_  [%RVE] | Overall | 55 | 6.50 ± 4.15 | 6.14 ± 4.69 | 6.61 ± 4.82 | 1.2968 | 0.2776 | - | - | - | - | - | - | - | - | - |
|  |  | Screwing | 55 | 7.61 ± 4.65 | 7.57 ± 5.20 | 8.39 ± 5.66 | 1.0827 | 0.3423 | - | - | - | - | - | - | - | - | - |
|  |  | Fastening | 55 | 7.62 ± 6.48 | 7.28 ± 7.92 | 7.64 ± 7.79 | 1.0391 | 0.3573 | - | - | - | - | - | - | - | - | - |
|  | RMS_90_  [%RVE] | Overall | 55 | 141.69 ± 57.20 | 133.85 ± 58.45 | 135.55 ± 56.22 | 1.7864 | 0.1725 | - | - | - | - | - | - | - | - | - |
|  |  | Screwing | 55 | 145.91 ± 58.78 | 139.35 ± 60.35 | 142.52 ± 59.35 | 0.9814 | 0.3781 | - | - | - | - | - | - | - | - | - |
|  |  | Fastening | 55 | 146.33 ± 69.01 | 122.52 ± 62.60 | 114.85 ± 58.87 | **14.6423** | **< 0.0001** | **23.82** | **0.0002** | **0.3726** | **31.49** | **< 0.0001** | **0.4922** | 7.67 | 0.1722 | 0.1236 |
|  | RMS_CV_ | Screwing | 55 | 0.92 ± 0.16 | 0.93 ± 0.16 | 0.94 ± 0.18 | 1.4962 | 0.2286 | - | - | - | - | - | - | - | - | - |
|  |  | Fastening | 55 | 1.25 ± 0.30 | 1.26 ± 0.34 | 1.25 ± 0.34 | 0.0844 | 0.9191 | - | - | - | - | - | - | - | - | - |
| M.  flexor  carpi  radialis | RMS_MEAN_  [%RVE] | Overall | 52 | 21.87 ± 15.24 | 21.49 ± 14.52 | 19.69 ± 16.42 | 2.3033 | 0.1051 | - | - | - | - | - | - | - | - | - |
|  |  | Screwing | 52 | 26.05 ± 21.29 | 26.47 ± 21.10 | 24.21 ± 23.05 | 1.4861 | 0.2311 | - | - | - | - | - | - | - | - | - |
|  |  | Fastening | 52 | 17.74 ± 15.60 | 16.74 ± 12.54 | 14.40 ± 9.38 | 2.6187 | 0.0778 | - | - | - | - | - | - | - | - | - |
|  | RMS_10_  [%RVE] | Overall | 52 | 4.26 ± 2.71 | 3.97 ± 2.07 | 3.75 ± 2.10 | 0.7165 | 0.4909 | - | - | - | - | - | - | - | - | - |
|  |  | Screwing | 52 | 6.42 ± 3.82 | 6.22 ± 3.20 | 5.72 ± 3.06 | 0.8225 | 0.4422 | - | - | - | - | - | - | - | - | - |
|  |  | Fastening | 52 | 6.74 ± 4.87 | 7.04 ± 4.87 | 6.29 ± 3.91 | 0.6529 | 0.5227 | - | - | - | - | - | - | - | - | - |
|  | RMS_90_  [%RVE] | Overall | 52 | 63.03 ± 64.65 | 63.65 ± 58.63 | 57.24 ± 58.08 | 1.0335 | 0.3594 | - | - | - | - | - | - | - | - | - |
|  |  | Screwing | 52 | 64.92 ± 67.81 | 66.33 ± 62.95 | 60.41 ± 62.29 | 0.6642 | 0.5169 | - | - | - | - | - | - | - | - | - |
|  |  | Fastening | 52 | 65.90 ± 80.80 | 61.36 ± 66.91 | 52.52 ± 53.75 | **4.0904** | **0.0196** | 4.54 | 0.7053 | 0.0333 | **13.38** | **0.0095** | **0.2316** | 8.84 | 0.0256 | 0.2082 |
|  | RMS_CV_ | Screwing | 52 | 0.70 ± 0.16 | 0.70 ± 0.16 | 0.72 ± 0.19 | 0.1758 | 0.8390 | - | - | - | - | - | - | - | - | - |
|  |  | Fastening | 52 | 0.90 ± 0.20 | 0.90 ± 0.25 | 0.89 ± 0.25 | 0.6141 | 0.5431 | - | - | - | - | - | - | - | - | - |

Supplementary Material 1. Continued.

| **Muscle / Body part** | **Parameter** | **Task** | **Subjects**  **N** | **Day 1** | **Day 2** | **Day 3** | **Main factor: day** | | **Post hoc tests** | | | | | | | | |
| --- | --- | --- | --- | --- | --- | --- | --- | --- | --- | --- | --- | --- | --- | --- | --- | --- | --- |
|  |  |  |  |  |  |  |  |  | **Day 1 vs. 2** | | | **Day 1 vs. 3** | | | **Day 2 vs. 3** | | |
|  |  |  |  | **Mean ± SD** | **Mean ± SD** | **Mean ± SD** | ***F*-value** | ***p*-value** | **MD** | ***p*-value** | **Cohen’s *d*** | **MD** | ***p*-value** | **Cohen’s *d*** | **MD** | ***p*-value** | **Cohen’s *d*** |
| M.  extensor  digitorum | RMS_MEDIAN_  [%RVE] | Overall | 56 | 45.29 ± 22.23 | 42.51 ± 23.57 | 40.46 ± 22.00 | **8.5609** | **0.0004** | **2.78** | **0.0164** | **0.1798** | **4.83** | **< 0.0001** | **0.2929** | 2.05 | 0.0962 | 0.1150 |
|  |  | Screwing | 56 | 50.34 ± 25.56 | 48.38 ± 28.66 | 46.00 ± 26.25 | **6.3243** | **0.0025** | 1.96 | 0.0586 | 0.1389 | **4.38** | **0.0006** | **0.2506** | 2.43 | 0.1034 | 0.1108 |
|  |  | Fastening | 56 | 34.76 ± 18.44 | 32.24 ± 16.46 | 31.76 ± 19.19 | **3.3087** | **0.0402** | 2.52 | 0.1630 | 0.1279 | **2.99** | **0.0115** | **0.2185** | 0.48 | 0.2468 | 0.1019 |
|  | RMS_10_  [%RVE] | Overall | 56 | 12.70 ± 7.06 | 10.13 ± 6.59 | 9.12 ± 5.84 | **18.3460** | **< 0.0001** | **2.57** | **< 0.0001** | **0.4581** | **3.57** | **< 0.0001** | **0.6485** | 1.01 | 0.0694 | 0.1980 |
|  |  | Screwing | 56 | 17.45 ± 9.45 | 15.03 ± 8.73 | 14.04 ± 8.07 | **13.7213** | **< 0.0001** | **2.42** | **0.0030** | **0.2781** | **3.41** | **< 0.0001** | **0.4350** | 0.99 | 0.0317 | 0.1894 |
|  |  | Fastening | 56 | 16.43 ± 9.30 | 15.60 ± 8.87 | 15.18 ± 10.42 | **3.5037** | **0.0335** | 0.83 | 0.4617 | 0.0651 | **1.25** | **0.0115** | **0.2057** | 0.42 | 0.0696 | 0.1519 |
|  | RMS_90_  [%RVE] | Overall | 56 | 83.00 ± 45.24 | 80.70 ± 53.23 | 75.60 ± 43.64 | **5.9647** | **0.0035** | 2.31 | 0.0060 | 0.1338 | **7.41** | **0.0008** | **0.2468** | 5.10 | 0.1139 | 0.1074 |
|  |  | Screwing | 56 | 84.22 ± 47.10 | 82.36 ± 55.66 | 77.29 ± 45.61 | **5.2413** | **0.0067** | 1.86 | 0.0938 | 0.1219 | **6.94** | **0.0016** | **0.2307** | 5.08 | 0.1250 | 0.1040 |
|  |  | Fastening | 56 | 84.14 ± 38.57 | 76.87 ± 32.94 | 76.82 ± 38.52 | **4.2677** | **0.0164** | 7.27 | 0.0360 | 0.1926 | **7.32** | **0.0060** | **0.2391** | 0.05 | 0.4999 | 0.0578 |
|  | RMS_CV_ | Screwing | 56 | 0.45 ± 0.11 | 0.45 ± 0.10 | 0.46 ± 0.12 | 2.1446 | 0.1220 | - | - | - | - | - | - | - | - | - |
|  |  | Fastening | 56 | 0.65 ± 0.20 | 0.65 ± 0.19 | 0.66 ± 0.21 | 0.1644 | 0.8486 | - | - | - | - | - | - | - | - | - |
| Heart | HR_MEAN_  [bpm] | Overall | 52 | 91.26 ± 15.29 | 87.60 ± 11.99 | 87.57 ± 11.49 | **5.9095** | **0.0037** | **3.66** | **0.0038** | **0.2683** | **3.69** | **0.0035** | **0.2759** | 0.03 | 0.9780 | 0.0029 |
|  |  | Screwing | 52 | 92.17 ± 15.42 | 88.41 ± 12.02 | 88.35 ± 11.23 | **9.3799** | **0.0002** | **3.76** | **0.0003** | **0.2737** | **3.82** | **0.0003** | **0.2865** | 0.06 | 0.9509 | 0.0054 |
|  |  | Fastening | 52 | 88.87 ± 15.39 | 84.98 ± 12.14 | 85.00 ± 11.52 | **6.3511** | **0.0025** | **3.89** | **0.0025** | **0.2827** | **3.87** | **0.0027** | **0.2874** | 0.02 | 0.9853 | -0.0020 |
| Forearm | ACC_MEAN_  [mm/s^2^] | Overall | 57 | 237.13 ± 56.34 | 218.65 ± 51.36 | 209.45 ± 49.66 | **13.6590** | **< 0.0001** | **18.48** | **0.0009** | **0.3432** | **27.67** | **< 0.0001** | **0.5222** | 9.19 | 0.0910 | 0.1820 |
|  |  | Screwing | 57 | 261.84 ± 65.51 | 244.35 ± 60.95 | 235.34 ± 58.49 | **9.0997** | **0.0002** | **17.49** | **0.0066** | **0.2766** | **26.50** | **< 0.0001** | **0.4274** | 9.01 | 0.1567 | 0.1508 |
|  |  | Fastening | 57 | 208.64 ± 68.02 | 199.90 ± 71.89 | 194.56 ± 71.02 | 2.7604 | 0.0676 | - | - | - | - | - | - | - | - | - |
|  | ACC_CV_ | Screwing | 57 | 0.74 ± 0.12 | 0.74 ± 0.12 | 0.75 ± 0.12 | 0.3793 | 0.6852 | - | - | - | - | - | - | - | - | - |
|  |  | Fastening | 57 | 1.52 ± 0.29 | 1.56 ± 0.34 | 1.61 ± 0.36 | **3.5898** | **0.0308** | 0.03 | 0.2627 | -0.1093 | **0.08** | **0.0087** | **-0.2514** | 0.05 | 0.1257 | -0.1345 |
| - | RPD | Overall | 57 | 2.12 ± 2.69 | 1.65 ± 2.33 | 1.50 ± 2.17 | 1.9850 | 0.1422 | - | - | - | - | - | - | - | - | - |
