# Supplementary figures and images for: The Role of Motor Learning on Measures of Physical Requirements and Motor Variability During Repetitive Screwing
Source: Int J Environ Res Public Health. 2019 Apr 6;16(7):1231. doi: 10.3390/ijerph16071231 (PMC6479693; doi:10.3390/ijerph16071231)

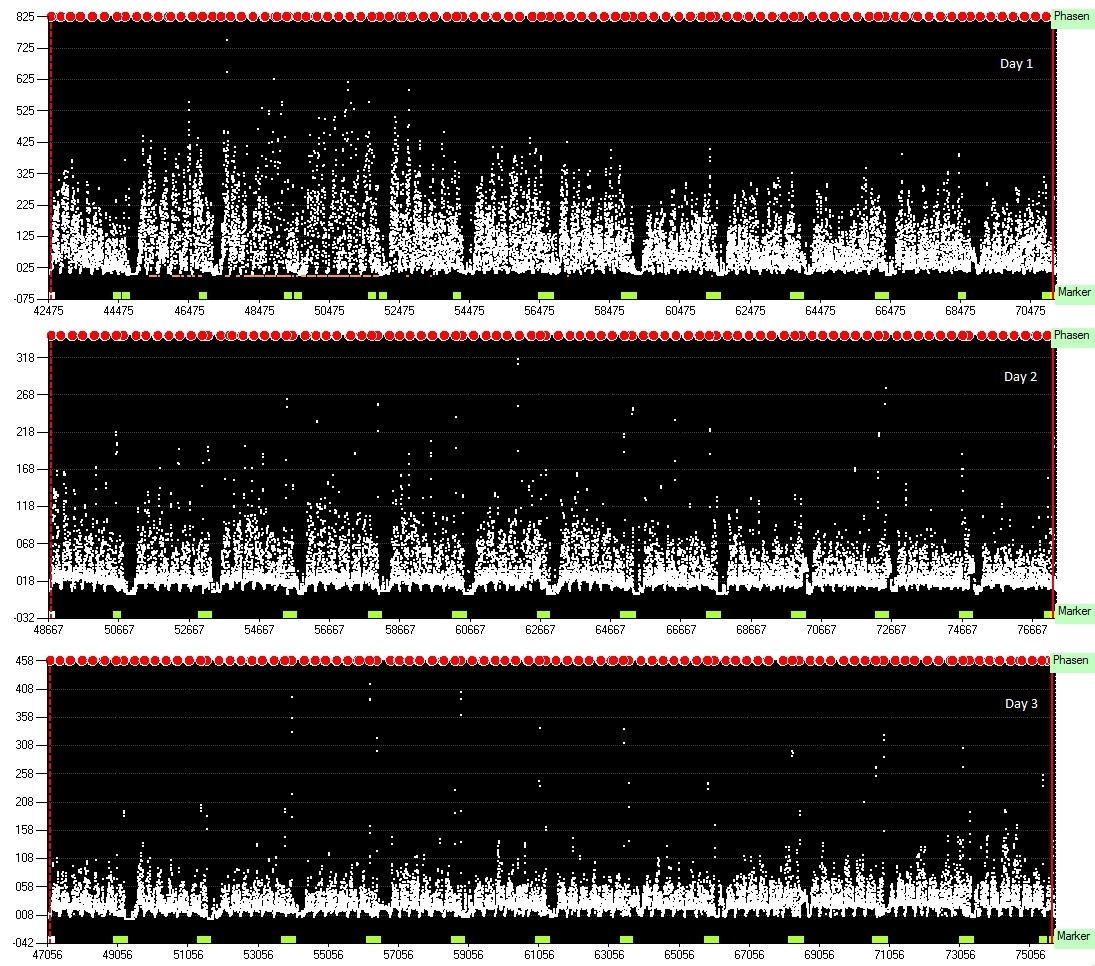

Supplement: Supplementary file 1 [file ijerph-16-01231-s001.zip › Figure_S_for_Supplementary_Material.jpg]
